# Noninvasive total counting of cultured cells using a home-use scanner with a pattern sheet

**DOI:** 10.1016/j.isci.2024.109170

**Published:** 2024-02-09

**Authors:** Mitsuru Mizuno, Yoshitaka Maeda, Sho Sanami, Takahisa Matsuzaki, Hiroshi Y. Yoshikawa, Nobutake Ozeki, Hideyuki Koga, Ichiro Sekiya

**Affiliations:** 1 Center for Stem Cell and Regenerative Medicine, Tokyo Medical and Dental University (TMDU), 1-5-45, Bunkyo-ku, Yushima, Tokyo 113-8519, Japan; 2 Medical & Healthcare Division, Dai Nippon Printing Co., Ltd., Tokyo, Japan; 3 Department of Applied Physics, Graduate School of Engineering, Osaka University, 2-1, Yamadaoka, Suita City, Osaka 565-0871, Japan; 4 Department of Joint Surgery and Sports Medicine, Tokyo Medical and Dental University (TMDU), Tokyo, Japan

**Keywords:** Optical imaging, Biotechnology, Machine learning

## Abstract

The inherent variability in cell culture techniques hinders their reproducibility. To address this issue, we introduce a comprehensive cell observation device. This new approach enhances the features of existing home-use scanners by implementing a pattern sheet. Compared with fluorescent staining, our method over- or underestimated the cell count by a mere 5%. The proposed technique showcased a strong correlation with conventional methodologies, displaying R^2^ values of 0.91 and 0.99 compared with the standard chamber and fluorescence methods, respectively. Simulations of microscopic observations indicated the potential to estimate accurately the total cell count using just 20 fields of view. Our proposed cell-counting device offers a straightforward, noninvasive means of measuring the number of cultured cells. By harnessing the power of deep learning, this device ensures data integrity, thereby making it an attractive option for future cell culture research.

## Highlights

• Cell culture techniques are done manually, lowering their reproducibility
• By harnessing the power of deep learning, this device ensures data integrity
• The new cell observation method correlates highly with conventional cell counting
• The error rate of the scanner-based cell observation method was within 5%

## Graphical abstract

**Figure:**
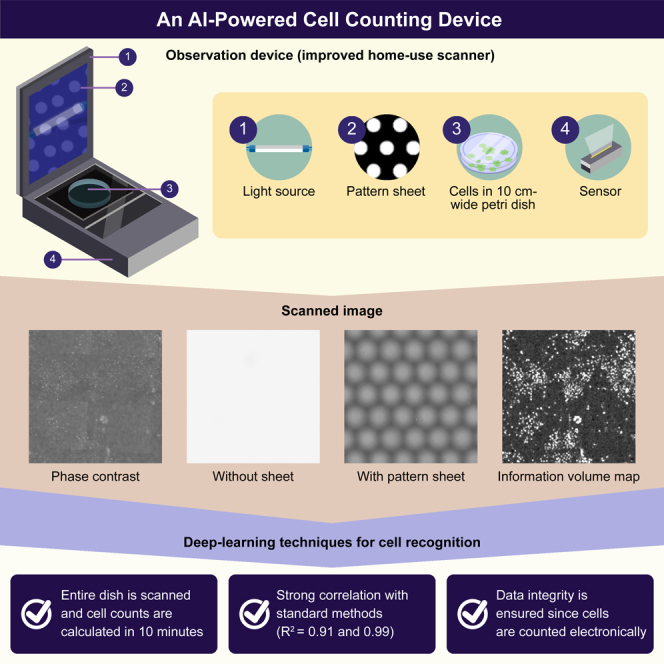

## Introduction

The reproducibility of scientific findings has become a topic of significant concern in recent years, particularly in the realms of fundamental and preclinical biological research.^1^^,^^2^^,^^3^ As the bedrock of scientific endeavor, research that lacks reproducibility squanders invaluable resources, time, and effort, all of which could be utilized in subsequent studies.^4^^,^^5^^,^^6^ Cell culture techniques, extensively employed in diverse contexts, such as new drug development and cell product technology, exhibit inevitable reproducibility variations, given that these processes are manually conducted. To alleviate these concerns, good manufacturing practice (GMP) compliance is deemed essential in the cell product manufacturing sector, serving to maintain traceability.^7^^,^^8^^,^^9^^,^^10^ Typically, cell count management is performed using invasive, mechanized cell-counting devices. However, establishing traceability throughout an entire operation proves challenging, as human involvement is frequently required for intermediate processes, such as the trypsinization of cells in a counting chamber. Various factors, such as variability in the preparative volume of a cell suspension, excessive pipetting, and inconsistent cell distribution in the dish, can detrimentally affect reproducibility. Additionally, the number of viable measurements is inherently limited owing to the resource-intensive nature of invasive cell collection, particularly in the case of sparse cell cultures, such as those observed in cell product processing.^11^^,^^12^

Consequently, our research aimed to develop a technology capable of addressing these challenges. Our objectives were two. The first was to enable the monitoring of the total cell count, thereby reducing the likelihood of human errors impacting reproducibility and traceability; the second, was to allow noninvasive measurements, thus minimizing the consumption or destruction of valuable cells. Various efforts have been expended to mechanize multiple operations to reduce human intervention.^13^^,^^14^ However, this aim remains partially unrealized owing to cost restrictions and the inability of these measures to address issues such as invasive cell consumption.

Noninvasive methods of observation, such as the continuous monitoring of cell proliferation, can be facilitated using commercially available systems. These systems typically feature an incubator box affixed to the motorized stage of an inverted microscope.^15^^,^^16^ However, while microscopes excel at observing small areas, they fall short in capturing a comprehensive view of culturable areas. Consequently, predicting the total number of cells within an entire dish based solely on these observations can introduce bias and may result in inaccuracies. Another noteworthy drawback of microscopy systems is their substantial cost, but these expensive, sophisticated lenses allow detailed analysis of morphological structures and protein expression related to cell function,^17^^,^^18^^,^^19^ which is important for quality control of cell products and is an unavoidable feature of the microscope’s structure. Advancements in several compact systems designed to be installed within incubators have been documented in recent years.^20^^,^^21^^,^^22^^,^^23^ Despite their affordability and design for continuous cell monitoring, their compact nature limits their ability to observe entire cell populations within culturable areas. Therefore, to date, there is no existing technology that facilitates the noninvasive measurement of total cell counts under traceable conditions.

To address these challenges, our technology was aiming to devise an instrument capable of quantifying the total cell count in a traceable, noninvasive manner. This instrument holds the potential for stable cell culture management in routine laboratory procedures, as well as for overseeing cell product production. It offers a specialized system for managing cell counts by observing the entire cell population within a culturable area. Our cell-counting technology comprises two components. The first component is an observation device that augments existing home-use scanners. This device utilizes a patterned sheet placed between a light source and the target object, which enables the visualization of cells on switching backgrounds. The second element of our technology is software trained to detect and enumerate all cells by analyzing unique cell image patterns. Furthermore, this process is conducted without human intervention; this allows cell count management to be solely overseen by electronic data management. In essence, our proposed system is a ground-breaking cell-counting device powered by artificial intelligence. This report presents an in-depth description of the system and its performance evaluation.

## Results

### Schema of scan imaging

**Figure 1:**
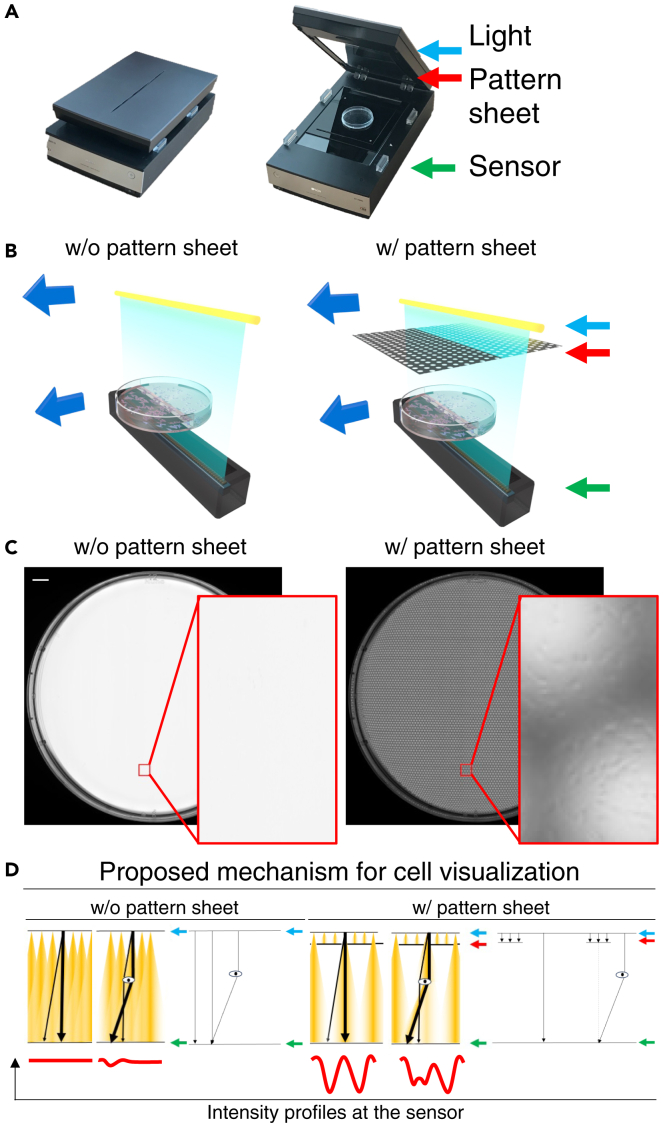
Schema of scan imaging (A) Overview diagram of scan imaging. The blue, red, and green arrows respectively indicate the position of the light, position of the pattern sheet, and position of the sensor, respectively. (B) Schema of scan imaging for cultured cells without a pattern sheet (left) and with a pattern sheet (right). (C) Scanned image of cultured cells without a pattern sheet (left) and with a pattern sheet (right). Scale bar is 1 cm. (D) Proposed mechanism for cell visualization without (left) and with a pattern sheet (right). The colored lines represent diffuse light from a white LED, and the black line extracts a representative optical ray. The center circle is assumed to be a cell. The red lines at the bottom of the figure represent intensity profiles at the sensor, and the right side of each figure presents the optical ray refracted at the cells.

In our developed imaging system utilizing a home-use scanner, the camera sensor was positioned to face the light source, while a patterned sheet was used to block the light source and create a shadow area (see Figures 1A and 1B). The cells could not be visualized without the pattern sheets (see Figure 1C, left), whereas with the pattern sheet, the cells were observable (see Figure 1C, right). We proposed the mechanism of cell visualization via a pattern sheet as described below. The diffused light source, which is the LED of the scanner, can be regarded as a summation of numerous point light sources. Then, without pattern sheet, the sensor receives numerous rays of light from all directions, and even if cells cause refraction to a part of light rays, the effect is relatively small and negligible, and cells may not be visualized (see Figure 1D, left). With the pattern sheet, the restricted light illumination produces inhomogeneous illumination onto cells. These rays of different intensities are refracted by the cells and reach different positions on the sensor compared to the absence of cells, respectively. Because the total amount of light reaching the sensor is suppressed by the pattern sheet, these changes will be large enough relative to each other to be observed as a pattern of shading that is different from the surrounding area (see Figure 1D, right). Furthermore, we confirmed the adaptability of this technology in various culture vessels beyond the 10 cm diameter dish (see Figure S1).

### Optical simulation model

**Figure 2:**
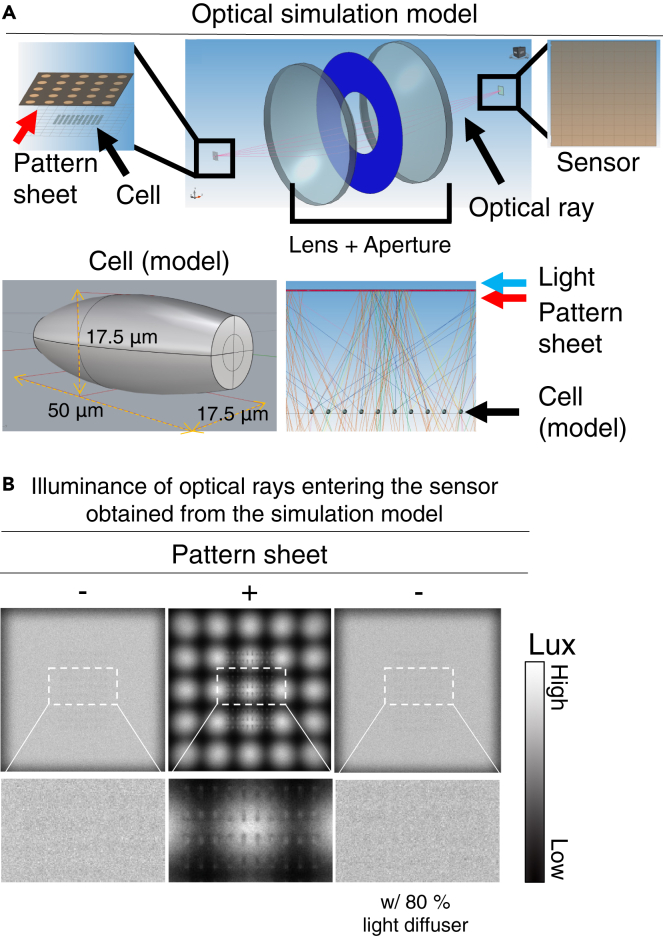
Optical simulation model (A) Optical simulation model for our scan imaging. LEDs (as general diffuse light sources), pattern sheets, and model cells were created for simulations (see further optical properties in Table S1). The transmitted optical rays were focused by a lens and observed by a charged-coupled device sensor. Model cells on the observation surface were prepared and lined up (numbers of cells was 10 × 10). The refractive index (RI) of model cells was set to 1.360 and the diffusion ratio of model cells was assumed to be 0.3. (B) Illuminances of optical rays detected by the sensor with and without pattern sheet and under 80% light-diffused conditions. Color scale bars denote illuminances (Lx: lm/m^2^). Detailed condition settings of the simulation model are shown in Table S1.

To investigate whether cell visualization can be achieved using pattern sheets, a simulation model was reconstructed based on the optical characteristics of a typical scanner, where diffused light is emitted from a light source and light is restricted by a pattern sheet (see Figure 2A). The detailed condition settings of the simulation model are presented in Table S1. Model cells were designed and evaluated to observe the type of light that would be detected by the sensor with and without the pattern sheet (see Figure 2A). The illuminances of the optical rays detected by the sensor with and without a pattern sheet are presented in Figure 2B. In the absence of the pattern sheet, the light transmitted through the model cells to the sensor is homogeneous without visualizing clear contrast of cells under this simulation (see Figure 2B, left). In the presence of pattern sheets, model cells refracted or reflected light rays with enhancing contrast of cells (see Figure 2B, middle). In the case of using homogeneous illumination (without pattern sheets) using an 80% light diffuser, no model cells were visualized (see Figure 2B, right).

### Cell detection by scanning

**Figure 3:**
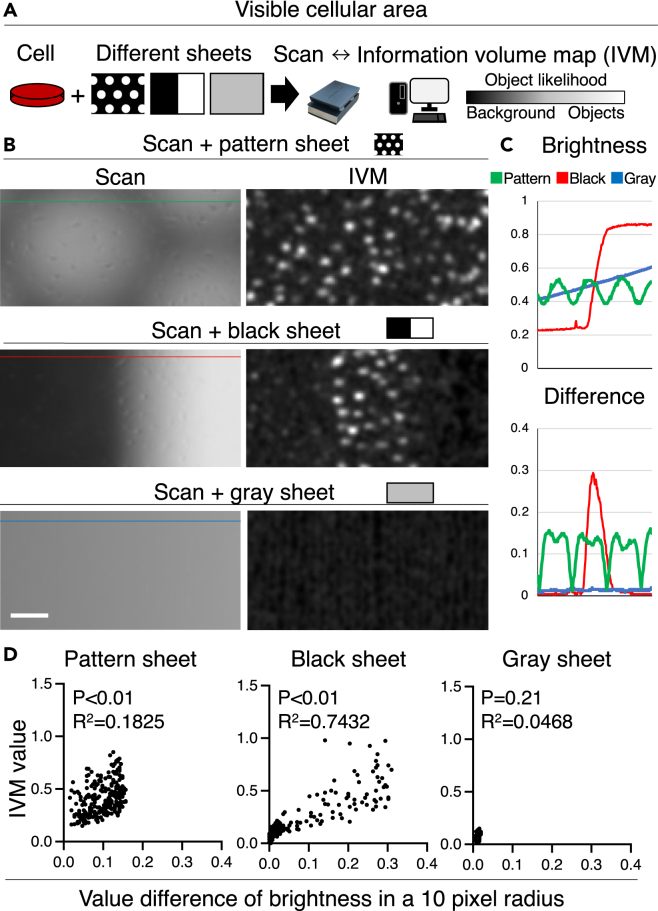
Cell detection by scanning (A) Schema of examination, and (B) scan image, and information volume map (IVM) image are shown. IVM images were visualized as objects from scanned images using patterned, black, and gray sheets. Scale bar is 100 μm. (C) Image brightness and differences in brightness in a 10-pixel radius for each image are presented along the y axis. The x axis represents the coordinates in the images. (D) Correlation diagram of the quantification of the IVM values in the form of object visibility and differences in brightness in regions with a 10-pixel radius; P and R values were calculated using Spearman’s correlation coefficient.

To determine the conditions under which cell visualization is possible, we performed scanning using multiple sheets. The cells were visualized in the scanned images with the pattern sheet, and the outcomes were quantified in the form of IVM values (see Figure 3A). Simultaneously, IVM values were calculated from scanned images obtained using a black sheet with significant brightness variation and a gray sheet corresponding to the brightness transition area after the black sheet (see Figures 3B and 3C). Consequently, we observed that the IVM values in the image increased when the difference in brightness within a 10-pixel radius became larger (see Figure 3C, left and center). Conversely, without the brightness transition, the objects could not be detected (see Figure 3C, right).

### Selection of pattern sheet based on the evaluation of scanned images

**Figure 4:**
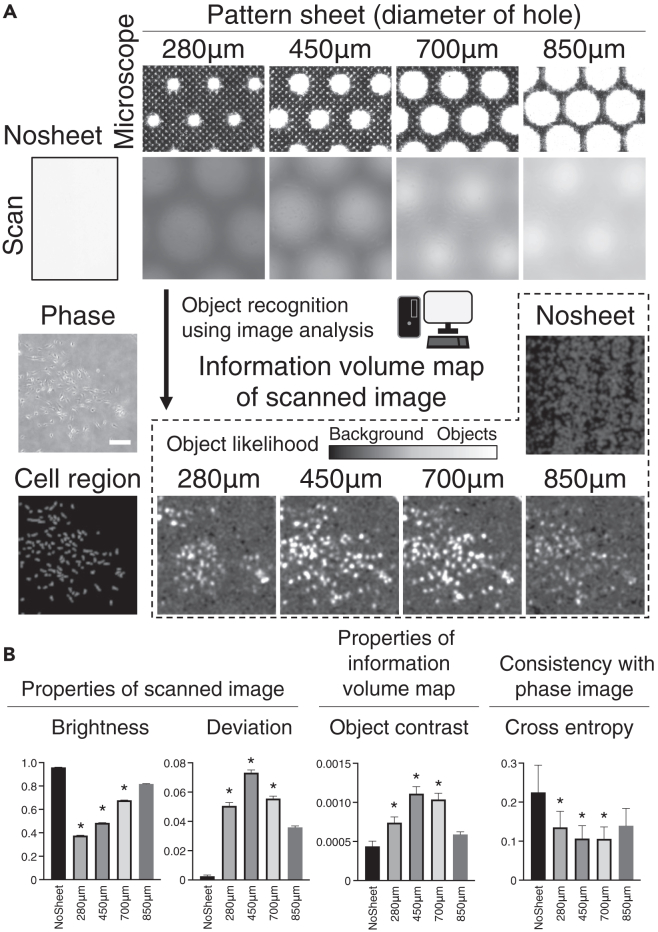
Selection of pattern sheet based on the evaluation of scanned images (A) Microscopic image of each pattern sheet scanned image for each pattern sheet and without the use of sheets, and phase image and cell region of the phase image (left). Information volume map of the scanned image after cell recognition using deep learning (right). The color bar represents deep-learning judgment results between cells and the background. Scale bar is 500 μm. (B) Properties of the scanned image in terms of brightness and deviation (left), and properties of information volume map derived from the scanned image in terms of contrast (middle). Evaluation of consistency between phase image and information volume map using cross-entropy (right). Data are presented as mean ± standard deviation (SD). ∗p < 0.05 for comparisons with the value at NoSheet using the Kruskal–Wallis test with Dunn’s test.

The range of cell visualization, measured manually from the image, was approximately 680 μm wide and was centered on the black-and-white transition of the sheet (see Figure S2A). The percentage of the area within the cell visualization range (ranging from the black-and-white transition to the entire pattern sheet), was calculated for four types of pattern sheets with hole sizes equal to 280, 450, 700, and 850 μm. The pattern sheet with 450 μm holes exhibited the highest coverage ratio of 96.7% (see Figure S2B). To demonstrate the theoretically optimal conditions, we scanned culture dishes with different pattern sheets (four different hole sizes) (see Figure 4A). Different scanned images were obtained for each pattern sheet (see Figure 4A). As the amount of light reaching the sensor was limited by the pattern sheets, the brightest scanned images were obtained when no pattern sheets were used, while the condition with the 280 μm hole pattern sheet produced the darkest image (see Figure 4B).

In terms of deviation, which reflects the brightness distribution in an image, the pattern sheet with 450 μm holes exhibited the highest deviation (see Figure 4B). An IVM was generated from the scanned images, where white areas represented potential objects and black areas represented potential background regions (see Figure 4A). The 450 μm hole pattern sheet yielded the IVM with the greatest contrast, thus indicating clear differentiation between objects and background (see Figure 4B). Cell regions were identified based on the phase images (see Figure 4B). The IVM derived from the scanned images and the cell region image (from the phase images) were compared, and the similarity of each image was quantified using cross-entropy (see Figure 4B). The 450 μm and 700 μm hole patterns yielded smaller values, thus indicating higher similarity and thus considered favorable patterns. Based on these findings, we selected the 450 μm hole pattern sheet for further analysis.

To assess the detection limit of this technology, we evaluated visibility using polystyrene microbeads. A comparative analysis using the IVM quantified the information content in scanned images and demonstrated detectable information in images with bead sizes equal to 5, 10, 20, and 30 μm. However, the 1 μm beads were too small to extract meaningful information from the image data. While the resolution limit was determined to be 5 μm, the areas in which beads were undetectable were confirmed manually, thus indicating that the stable visible size using the current equipment configuration was 10 μm or larger (see Figures S3A and S3B).

### Accuracy of scan imaging

**Figure 5:**
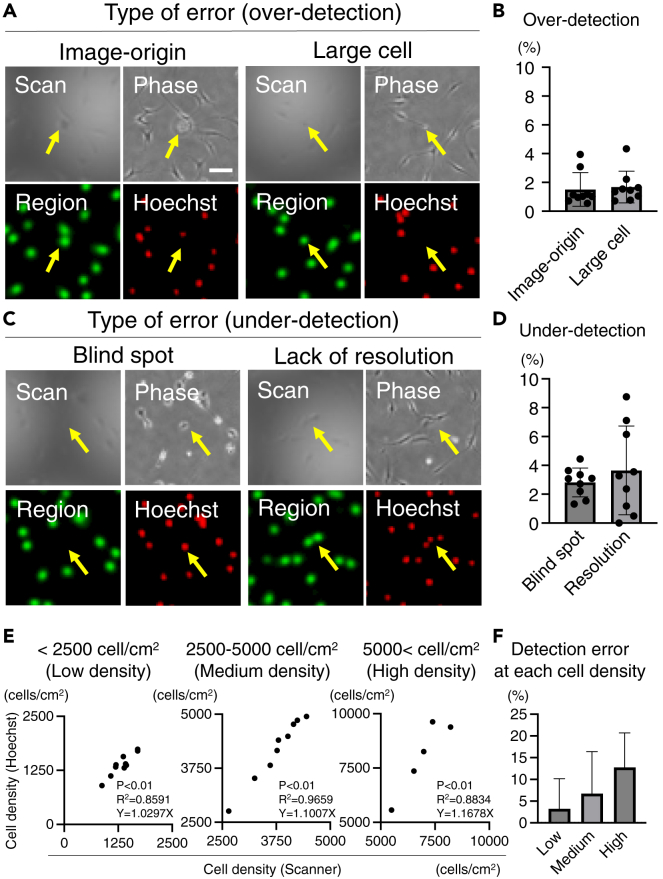
Accuracy of scan imaging (A) Types of error associated with overdetection. Representative images for error types of image origin (left) and large cells (right). The scanned image (Scan) and phase represent a raw image. Image of the cell region (Region) and Hoechst-stained image shown in pseudo color. Yellow arrows indicate the differences between each image. Scale bar is 100 μm. (B) Percentage of each error identified in nine images. Data are presented as mean ± SD. (C) Types of errors associated with under-detection. Representative images for error types of the dead angle (left) and lack of resolution (right). (D) Percentage of each error identified in nine images. Data are presented as mean ± SD. (E) Accuracy at each cell density from low to high density. (F) Detection error at each density. Data are presented as mean ± SD.

To assess accuracy, phase, Hoechst-stained, and scanned images of the same field of view were acquired and compared. Errors resulting in different detection outcomes compared with the conventional method were classified into two categories: overdetection and underdetection (see Figure 5). Overdetection errors caused by debris or detached cells during staining, referred to as image-origin errors, accounted for 1.51 ± 1.17% compared with the number of nuclei detected in the Hoechst-stained images (see Figures 5A and 5B). Another type of overdetection error, known as large cell-derived errors, occurred when large cells were detected as two cells; this resulted in errors equal to 1.68 ± 1.10% (see Figures 5A and 5B). Underdetection errors, referred to as blind spots, were mainly observed in dark areas of the shading pattern and contributed to an error of 2.82 ± 1.00% (see Figures 5C and 5D). Lack of resolution, another type of underdetection error, occurred in densely populated areas and resulted in an error of 3.65 ± 3.70% (see Figures 5C and 5D). The accuracy at each cell density was determined by comparing the cell density calculated using Hoechst-stained images as the reference value (mother number) with that detected by the scanner (number of children). The accuracies for low, medium, and high densities were 3.22 ± 6.91%, 6.72 ± 9.67%, and 12.8 ± 7.94%, respectively (see Figures 5E and 5F).

### Comparison to conventional methods

**Figure 6:**
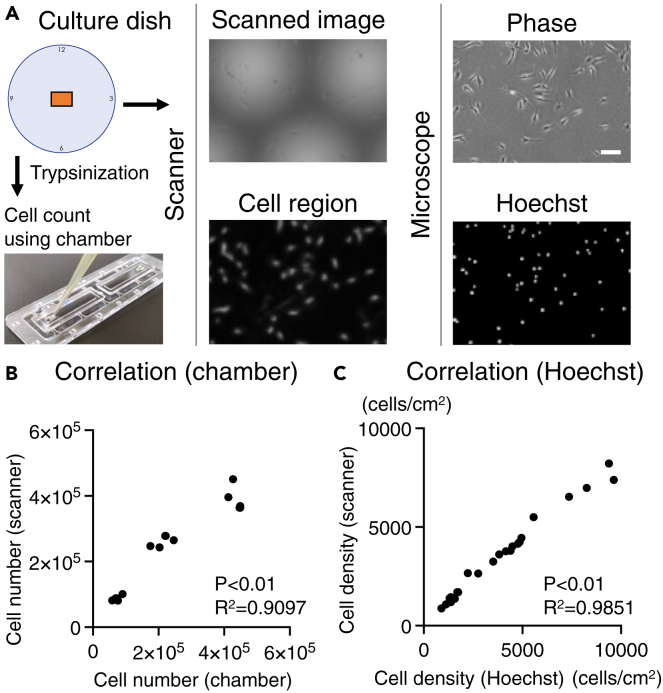
Comparison to conventional methods (A) Methods of comparison between scanned image cell counts and cell counting using a chamber or Hoechst-stained images. Scale bar is 100 μm. (B) Correlation between cell number in the entire dish and cell count using a chamber (n = 15). (C) Correlation between scanned image and Hoechst-stained image. Comparisons with Hoechst-stained images were performed based on cell density (cells/cm^2^) calculated from 5 × 5 fields of view at the same location in a scanned image (n = 25).

The cell counts in the scanned images were verified and compared with those obtained using the conventional method (see Figure 6A). The total cell number calculated from the scanned image of the entire dish was analyzed and correlated with the total cell number measured using the chamber method. The cell density calculated from the scanned image of the same area as that of the Hoechst-stained image was analyzed for correlation with the Hoechst-stained image obtained from a tiled image of approximately 1.5 cm^2^ captured with a microscope. For the chamber method, an R^2^ value of 0.91 was obtained (see Figure 6B), while for the Hoechst-stained image, an R^2^ value of 0.99 was obtained (see Figure 6C).

### Usefulness of whole-dish imaging

**Figure 7:**
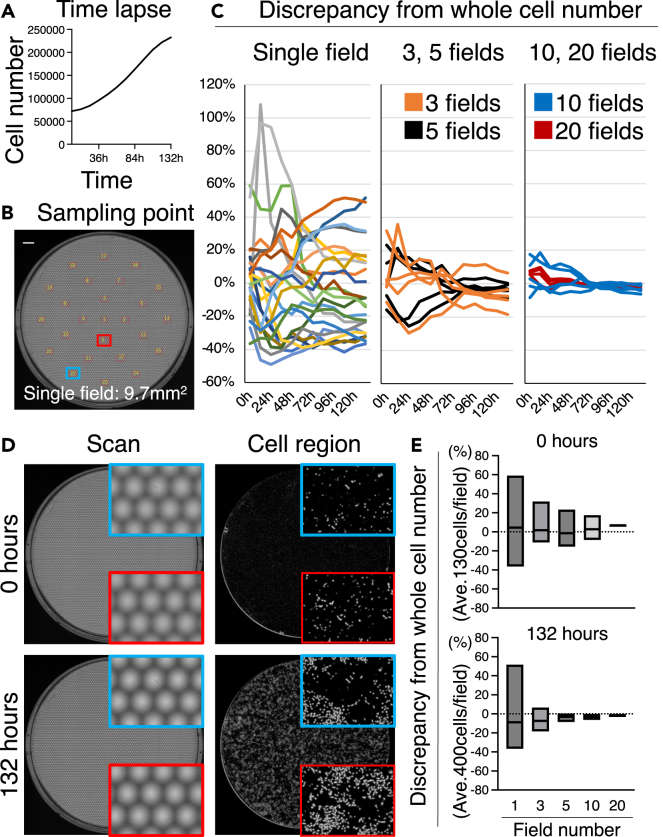
Usefulness of whole-dish imaging (A) Time evolution of the cell number calculated from time-lapse scanned images. (B) Locations of 25 arbitrarily extracted pseudo-microscopic fields of view and area of the captured images (mm^2^). Scale bar is 1 cm. (C) Discrepancy compared with the total cell number calculated from scanned images. The total cell number calculated from the scanned image was 0%. The horizontal axis represents elapsed time. Individual fields are presented with data from 25 fields of view (left). For the groups of three and five fields, the graph presents five average values randomly selected from 25 fields of view (middle). For the groups of 10 and 20 fields, the graph presents five average values randomly selected from 25 fields of view (right). (D) Scanned images and cell regions captured at 0 and 132 h. Representative locations are highlighted by blue and red squares, respectively. (E) Discrepancy between the number of cells in each field of view and the total number of cells at 0 and 132 h. Data are presented as maximum to minimum and average values.

To evaluate the time-lapse observations of the scanner and understand the variation in cell density depending on the observation position, a comparative simulation was conducted. Time-lapse imaging was performed to track the number of cells over time (see Figure 7A). From the time-lapse scan images, a single field of view and sets of 3, 5, 10, and 20 fields of view were extracted from the 25 fields (with the same size) as the microscopic images (see Figure 7B). The discrepancy in cell density was calculated as the same area was observed for 6 days (see Figures 7C and 7D).

When estimating based on a single field of view, the maximum error exceeded +100% at low cell densities, and even at high cell densities, the error was ±40% (see Figure 7C). Observations with three fields of view showed errors of ±30% at low densities and up to −20% at high densities (see Figures 7C and 7E). With five and 10 fields of view, the errors were +30% to −20% at low densities; reduced errors were obtained at high densities (see Figures 7C and 7E). The errors observed with 20 fields of view indicated that the predicted cell density could be approximately 10% higher at low densities, but exhibited no discrepancy at high densities (see Figures 7C and 7E).

## Discussion

In this study, we developed a cell observation device using a home-use scanner. This device enables the measurement of cell numbers by analyzing cell-specific image patterns with a trained algorithm. This method offers a simple and noninvasive approach to count accurately the total number of cultured cells while maintaining data integrity through digital management. By applying image recognition technology, cell-like objects with similar functions to those of the human eye can be identified. In other words, our cell-counting device operates solely based on artificial intelligence algorithms. The accuracy of this method is comparable to conventional chamber and fluorescent staining methods. Problems with conventional technology, including concerns regarding the stability and reliability of the counting process, persist owing to the manual nature of detachment and dilution steps in cell suspensions. In contrast, our proposed device offers a simple and cost-effective solution for cell counting. Furthermore, the device allows for the simultaneous observation of an entire dish, eliminating the bias often introduced in microscopic observations. Its implementation is cost-effective, as it utilizes a home-use scanner. However, it is important to note that the proposed device does not provide cell morphology information but specializes in counting cells accurately.

Even though the optical simulation model successfully enabled us to visualize model cells under pattern sheets, we implemented essential optical conditions of the scanner due to limitations in simulation implementation. Furthermore, we confirmed that model cells could not be visualized under simple light-districted conditions. The results of the simulation model and the actual imaging were comparable. Cell counting using our proposed technique is similar to the principle of the dark field microscopy or the Schlieren method; however, the detailed mechanism underlying our methods has not been clarified. The dark field microscopy method is a technique to obtain high-contrast images irradiating from an oblique angle and observing the scattered light by the sample.^24^ In our system, pattern sheet restricts diffused light originating from LED, to produce a conical-shaped area with an intensity gradient from the center toward the edge. Then, such refracted light at the cells reaches the shaded region, leading to increase the signal into the sensors. Our cell visualization technology may resemble the principle of dark-field microscopy in that it detects light scattered by cells. While, the Schlieren method is an optical technique that uses changes in the direction of light rays to make the imaged part visible when parts of the transparent body possess different refractive indices.^25^ Our method can be explained according to these principles by considering cells as regions with different refractive indices in the culture medium.^26^ In other words, cells cause light refraction, altering the position of light reaching the scanner’s sensor. Because these changes are small, it can be assumed that in the absence of a pattern sheet, these differences would be obscured by the diffusion of bright light from the LED, rendering observations impossible. The scanned cells with pattern sheet may be visualized by a complex combination of these principles, however, detailed optical analysis is needed to elucidate the cell visualization principles of our scanning system.

In our study, we noted significant variations in the identification of cellular areas when selecting different hole sizes for the pattern sheet. The selection of an optimal pattern that yields precise cell representation is a crucial aspect of our technology. We chose a pattern sheet with 450 μm holes, striking a balance between human perception, artificial intelligence capabilities, and industrial production feasibility. Our system achieves cell visualization through the contrast between black and white, as demonstrated by the invisibility of cells when imaged with similar brightness levels. According to mathematical calculations using the virtual visualization area, a 450 μm patterned sheet is most effective in creating frequent 680 μm zones of black and white transitions. While the specific optical mechanisms behind this remain unclear, the 450 μm size appears to be most effective in replicating the visible transition zones. However, considering our technology’s reliance on artificial intelligence rather than human vision, investigating patterns specifically optimized for AI could be beneficial. Exploring various patterns, including squares, triangles, or their combinations, may lead to identifying more suitable patterns. Furthermore, given that the cell size in this study was around 15 μm, different patterns may be optimal for various cell types. The effectiveness of these patterns could also differ for floating cells, clustered cells, or spheroids. Future research should therefore include testing with multiple cell types, ensuring the broad applicability and versatility of our technology.

The proposed technique demonstrated accuracy with an error ≤5%, with slight over- and undercounts relative to the number of cells counted based on fluorescent staining. Although an error of approximately 10% is anticipated in high-density scenarios, which is uncommon in normal cultures, this error could be mitigated through in silico correction utilizing a discernible pattern. By utilizing artificial intelligence for cell counting, the proposed device can effectively prevent overdetection resulting from image stains using additional training. Moreover, it can address underdetection caused by insufficient resolution by enhancing the camera resolution through mechanical means. Despite the presence of a small margin of error, the cost-effective nature of cell counting using this method offers significant advantages, thus rendering this approach valuable for various practical applications.

The measured accuracy of the proposed method achieved an R^2^ value of 0.99 when compared with the cell count obtained via fluorescent staining, thus surpassing the R^2^ value of 0.91 associated with the chamber method. However, unlike the conventional chamber method, this technology does not allow the distinction between live and dead cells, as observed with other microscopes. Regardless, the proposed technique can prevent the blurring caused by human interference. The key advantage of mechanization lies in ensuring traceability and reliability. Although the automation of cell production holds promise across many fields,^27^^,^^28^^,^^29^ it has not materialized yet owing to the absence of benefits that can offset the drawbacks of high cost. To comply with GMP standards and guidelines, such as the Code of Federal Regulations (Title 21 Part 11) “Compliance for Cell-counting Equipment,”^30^^,^^31^ exist to safeguard electronic data. However, data traceability remains limited as cell counts are ultimately obtained manually, and manipulations during dilution can affect the accuracy of cell counts. The proposed device can provide exceptional traceability and reliability by directly counting cells in cell culture dishes, thus making it highly advantageous for GMP compliance, even in settings involving human intervention.

The proposed technology enabled time-lapse imaging and eliminated the need for an expensive motorized stage typically required for wide-area, time-lapse microscopic imaging. With the proposed technique, capturing the entire dish became effortless, which implies that even if density variations occurred within a dish, the proposed technique has the potential to cover the entire dish, thereby eliminating errors. Conversely, microscopes have a limited field of view, necessitating the capture of multiple fields of view. To determine the number of fields of view needed to estimate the cell count of an entire dish using a microscope, we conducted simulations leveraging the comprehensive data captured by the proposed technique. The simulations revealed that a single field of view could potentially miscount cells with a maximum error of +100% with respect to the actual cell count. However, as the number of fields of view increased, the error decreased. Furthermore, the error tended to be higher in areas with low cell density and lower in areas with high cell density. This instrument sheds light on the previously (uncertain) technical challenges associated with microscopic observations.

In summary, we successfully developed a cell-counting device that utilizes a scanner to measure effortlessly and noninvasively the total number of cultured cells, thus eliminating the need for human involvement. This device achieves accuracy comparable with traditional methods while enabling simultaneous observations of the complete cell count within an entire culture dish. By utilizing deep-learning techniques and ensuring data integrity, our device shows great promise for diverse cell culture applications.

### Limitations of the study

It is important to acknowledge the study’s limitations. First, the cells utilized in this research were approximately 15–20 μm in size. Therefore, further technical validation was necessary to measure smaller cells or cells densely packed in colonies, such as inducible pluripotent stem cells.^15^ In experiments testing detection limits with micro-particles, particles as small as 5 μm were partially detectable. However, due to the camera’s 10 μm/pixel resolution, only fragmentary images were obtained, and detection was sporadic. Theoretically, measuring smaller cells could be feasible by upgrading the scanner’s camera and improving image resolution, which is an aspect we plan to explore through mechanical advancements. Second, a significant limitation pertains to the technical constraints of our optical simulation models. While the model adeptly visualizes cells, it does not perfectly replicate scan imaging. For example, the pattern sheet in the simulation is represented by a simplified module, essentially a black sheet with apertures. The actual pattern sheet, composed of polyester, possesses distinct absorption characteristics and a specific refractive index. Polyester’s light absorbance, particularly in the UV spectrum around 200–350 nm,^32^ diverges from the 400–800 nm wavelength range of white LEDs. Hence, we posited negligible impact on cell visualization. Nonetheless, the refractive index of polyester, ranging from 1.48 to 1.62, might have influenced the LED light as it penetrated the cells.^33^ However, according to Snell’s law, which suggests that the angles of incidence and emergence align in thin films, the impact of the sheet material was presumed minimal.^34^^,^^35^ However, it is imperative to recognize that the simulation model does not exactly replicate the scanning process; therefore, the principles underlying the simulation model and scan imaging might differ. Additionally, the optical principles involved in cell visualization within this study remain uncertain. Although successful visualization was achieved in the optical simulation model, a detailed optical analysis is essential to elucidate these principles. For instance, the optimal pattern size was different between the 10 cm dish and the T225 flask as a result of their different heights as culture vessels. A research environment facilitating multiple verifications of various optical factors will be necessary.

## STAR★Methods

### Key resources table

**Table:** 

REAGENT or RESOURCE	SOURCE	IDENTIFIER
**Biological samples**		

Synovial stem cells	Human	N/A

**Chemicals, peptides, and recombinant proteins**		

Collagenase	Sigma–Aldrich	C9263
α-minimum essential medium	Thermo Fisher Scientific	12571063
antibiotic–antimycotic	Thermo Fisher Scientific	15240062
fetal bovine serum	Thermo Fisher Scientific	A5256701

**Deposited data**		

Original code	This paper	https://doi.org/10.17605/OSF.IO/BSAR8

**Software and algorithms**		

MATLAB (versions 2020, 2021, and 2022)	MathWorks Inc	https://matlab.mathworks.com

### Resource availability

#### Lead contact

Further information and requests should be directed to and will be fulfilled by the Lead Contact, Dr. Mitsuru Mizuno (mizuno.arm@tmd.ac.jp).

#### Materials availability

This study did not generate new unique reagents.

#### Data and code availability

• The microscopy and scan image data reported in this study will be shared by the lead contact upon request.
• The original source code in this study is available on Open Science Framework. DOIs are listed in the key resources table.
• Any additional information required to reanalyze the data reported in this work paper is available from the lead contact upon request.

### Experimental model and study participants details

#### Human subjects

This study received approval from the Medical Research Ethics Committee of the Tokyo Medical and Dental University (M2017-142). All participants provided informed consent.

Although all Japanese female cells were used in this study, the race and sex differences in cell morphology does not affect the ability to recognize cells.

Human synovium was procured from the knees of three female donors (ages: 63–76) who were diagnosed with osteoarthritis and underwent total knee arthroplasty.

#### *In vitro* culture conditions

The synovial cells were cultured following previously established protocols.^36^ The synovium was digested using a solution of 3 mg/mL collagenase (Sigma–Aldrich Co., LLC, Merck KGaA, Darmstadt, Germany) at 37°C. After 3 h, the digested cells were filtered through a 70 μm cell strainer (Greiner Bio-One GmbH, Frickenhausen, Germany) and washed with phosphate-buffered saline (Thermo Fisher Scientific, MA, USA). The cells were cultured in a 10 cm dish (Nunc, Thermo Fisher Scientific) using α-minimum essential medium (α-MEM; Thermo Fisher Scientific) supplemented with 1% antibiotic–antimycotic (Thermo Fisher Scientific) and 10% fetal bovine serum (Thermo Fisher Scientific) in a cell culture incubator (Astec Co., Ltd., Fukuoka, Japan) at 37°C in a 5% CO_2_ atmosphere. To evaluate the culture vessel, cells were seeded in six-well plates (BD Falcon, NJ, USA), a dish with a 15 cm diameter (Corning by Thermo Fisher Scientific), T75 flask (Sumitomo Bakelite Co., Ltd., Tokyo, Japan), and T225 flask (Sumitomo Bakelite Co.). Each cell was ruled out for mycoplasma infection by PCR.

### Method details

#### Cell imaging

The images for this study were captured at a resolution of 2400 dots per inch (dpi) using a consumer-grade scanner (Model: GT-X980, Seiko Epson Corporation, Nagano, Japan) (see Figure 1A). This study presents data obtained using five instruments with different serial numbers. The scanner's camera resolution is 10 μm/pixel. Pattern sheets were fabricated by printing on a transparent overhead projector film with a thickness of 0.125 mm (Model BG-72P, Folex Coating GmbH, Köln, Germany) using a laser printer (Model Apeos C5570; Fujifilm Co., Tokyo, Japan). The material of the pattern sheet is polyester, characterized by a refractive index ranging from 1.48 to 1.62. Notably, it exhibits absorbance in the invisible UV spectrum, specifically within the 200-350 nm wavelength range.^32^^,^^33^^,^^37^ Scan imaging is presented in Video S1.

Video S1. Scan imaging, related to Figure 1 Video played at 20× speed for scanning the 10 cm dish.

Phase and Hoechst images were procured using a microscope (BZ-X700; Keyence Co., Ltd., Osaka, Japan).

#### Optical simulation model for scan imaging

Optical simulation model was constructed to verify how optical rays visualize cells. Optical simulation analysis was performed using Lighting Simulator CAD (Camerium Inc., Tokyo, Japan). Detailed settings for the simulation model are presented in Table S1. In brief, a white light-emitting diode (LED) light and a sensor were used for scanning. The optical rays transmitted through the model cell were focused by a lens to be collected by light to a charge-coupled device (CCD) sensor for observation.

The software visualized the illuminance of the simulated optical rays detected by the sensor.

#### Image analysis

For image analyses, programming was executed in MATLAB (versions 2020, 2021, and 2022, MathWorks Inc., Natick, MA, USA). Various indices were calculated for 10 randomly selected fields of view (500 × 500 pixels) from the scanned images.

The means of all pixel values (i.e., luminance values) in the scanned images were computed (utilizing the “mean” function in MATLAB) for various attributes of the scanned images, such as visibility and brightness. This mean served as an index for the overall brightness of the image. The variances of all pixel values (luminance values) within scanned images were quantified using the “std” function in MATLAB. This standard deviation estimate served as an indicator of the distribution of luminance brought about by the pattern sheet.

Regarding data retrievability properties, an information volume map was created using a basic procedure to estimate the likelihood of object regions within an image. The object contrast, which is the variance of all pixel values (object likelihood) of the information volume map, was quantified as an index of the ease of differentiating objects from the background. The shading pattern generated by a pattern sheet typically demonstrated a gradual transition in lightness and darkness across a large area, whereas the shading resulting from an object (typically from cells but occasionally from dust) exhibited rapid changes in lightness and darkness over a smaller area. We considered the gradual changes in lightness and darkness over a large area as the background. By calculating the magnitude of difference in comparison to the background pattern, we defined an information volume map as an image from which areas derived from objects could be extracted.

An input scanned image was blurred (using the “imgaussfilt” function in MATLAB with a σ parameter of 1.0) to generate a basic background image. A positive information map was generated by subtracting the background image from the original scanned image, truncating any negative values, and applying Gaussian blurring (using the “imgaussfilt” function in MATLAB with σ = 1.5). A negative information map was created by subtracting the original scanned image from the background image, truncating any negative values, and applying Gaussian blurring with σ = 1.5.

The information volume map was defined as the sum of both the positive and negative information volume maps. As the values derived from this computation were extremely small; values larger than 1.0 (pure white) were rounded down after amplification. This amplification was performed by multiplying values by 200 when displaying an image, and by 100 when calculating object contrast.

To maintain consistency between the phase image and information volume map, we evaluated the similarity of data utilizing cross-entropy. This measure indicates the level of difficulty in predicting one side's information (cell likelihood value from the phase image) solely based on the other side (object likelihood value from the information volume map).^38^^,^^39^ The cell regions in phase contrast images were analyzed using a combination of image processing techniques: morphological processing (using MATLAB’s “imtophat” function with radius 1 disk-type structured elements generated by the “strel” function), Gaussian blurring (using MATLAB’s “imgaussfilt” function, σ = 0.75), and a blend of minor area removal process (using MATLAB’s “imbinarize” function in conjunction with the “bwareaopen” function, parameter value = 2), and enhancement process (via MATLAB's “power” function, parameter value of 0.15). The higher the similarity between an information volume map and the cell region in the corresponding phase image, the lower the cross-entropy value, thus indicating a greater ease in correctly identifying cell regions in the scanned image.

#### Selection of pattern sheets

The diameter of the pattern sheet was selected based on the characteristics of the scan images, IVM, and microscope images. Scan images were evaluated from brightness and deviation. The characteristics of the IVM generated from the scanned image were analyzed by object contrast. The consistency with the phase contrast image captured with a microscope was evaluated by cross-entropy after the coordinates were manually aligned.

To evaluate the efficiency of the black-and-white switching of the pattern sheet, based on the value of 340 μm calculated from the experimental results, that is half the size of 680 μm, a virtual visualization area was generated around the black-and-white switching area of the pattern sheet, and the coverage to the total area was calculated.

#### Visibility of cells during scanning

Several sheets were utilized for scan imaging, and cell visibility was evaluated using different sheets. Each sheet was scanned at the cell locations within the same culture dish. Cell visibility was assessed using the information volume map of the scanned images to identify areas in which cells were visible. Variations in visibility across different sheets were quantified using image data based on brightness and the changes in brightness within a 10-pixel radius.

To establish the detection limit size, we used polystyrene microbeads ranging from 1 to 30 μm in size (Micromod Partikeltechnologie GmbH, Rostock, Germany) for evaluation. The detectability of these microbeads was assessed according to the differences in values between the background and objects within the information volume map (IVM).

#### Deep learning for cell recognition in scanned images

Our process of training a deep-learning model for cell recognition from scanned images consisted of two main phases: the training and the inference phase.

In the training phase, the model learns using labeled data, whereas in the inference phase, the model applies the learned patterns to identify cell regions in new images. To initiate the training phase, we first generated training data using phase, Hoechst-stained, and scanned images from the same dish, with cell images aligned visually based on the position and rotation angle. The regions representing cells and nuclei were identified based on a combination of image processing, visual inspection, and manual adjustments, producing numerous scan image pairs, cell region images, and nuclear region images.

Subsequently, we implemented deep learning for training, using scanned images as the input and training the model to identify cell regions. We used the Microsoft Cognitive Toolkit as our deep learning framework and the U-Net model. The model’s input and output sizes were set at 256 × 256 pixels. Training data were augmented by rotating and varying the brightness; we used Adam as the optimizer, the minibatch size was set to 64, learning rate was set to 0.1, and the inertia was 0.8. Some minor manual adjustments were also made, including the random variations of the brightness of the training data and temporal increase of the learning rate. This process culminated in an initial model capable of identifying cell regions from scanned images.

To improve the model's accuracy, we generated the training data for a second time. Scanned images captured under varying conditions, such as diverse cell densities and condensation, were processed using the initial model. Recurring errors, including “over-detection of cells in similar areas,” were rectified using image processing, while other errors were manually corrected to define cell regions. The rectified images were then used to create new pairs of scanned, cell region, and nuclear region images, thus forming new training data. These data were then used for additional training to refine the model for enhanced accuracy and stability.

The training phase was completed after four iterations of this additional training process and the final version of the model was trained. The final model demonstrated an accuracy of 97.3% after training using 1024 pairs of test data that varied in seeding density, culture elapsed time, condensation state, pattern sheet displacement, and media volume.

During the inference phase, the trained model was applied to new scan images for cell recognition. Cells were identified as clear white areas, whereas black areas were considered the background. In the gray areas, it was not readily apparent if they were cells or background, necessitating further investigation to ascertain their identity.

#### Error detection in scanned images

Discrepancies in scanned images were spotted by comparing nine randomly selected images from the full-dish scans with phase and Hoechst-stained images from identical locations. Cell regions in each scanned image were autonomously detected using the trained model, while nuclear regions were pinpointed via Hoechst staining. These images were manually compared using the software GNU image manipulation program (GIMP, version 2.10.32) to verify the correspondence between each cell and nucleus. Any discrepancies were classified based on a comparison with the original cell and nucleus. To evaluate accuracy across various cell densities, the cell density ascertained by Hoechst staining served as the reference population. The cell density detected by the scanner was considered as the sample population and percentage outcomes were expressed as mean ± standard deviation (SD).

#### Comparison to conventional methods

The entire surface of a 10 cm dish was scanned and processed using the trained model. After scanning, the dish was stained with Hoechst 33342 (Dojindo, Kumamoto, Japan), and images covering 5 × 5 fields of view (each field spanned 640 × 480 pixels) were acquired using phase contrast and fluorescence, and subsequently compiled into a tiled image. The same fields of view captured in the tiled images were extracted from the scanned image. The number of cells detected in the extracted area was divided by the area of the extracted image to calculate the cell density (n = 25 fields of view). Similarly, the count of nuclei detected in the tiled Hoechst-stained images was divided by the area of the tiled images to determine the cell density by Hoechst staining (n = 25 fields of view). Following the scanning and microscope imaging processes, cells were removed from the dish using trypsin-ethylenediaminetetraacetic acid (Thermo Fisher Scientific), and the resulting cell count served to define the cell density by chamber (n = 15 dishes).

The cell suspension in 1 mL of phosphate-buffered saline was pipetted gently multiple times. For analysis, 18 μL of the suspension was mixed with 2 μL of the staining compound. Cell numbers were then counted using Luna-FL (Logos Biosystems, VA, USA). Double staining with acridine orange for live cells and propidium iodide for dead/apoptotic cells was performed using a live/dead assay kit (Logos Biosystems).^40^ The obtained cell counts were corrected for dilution concentration to calculate the total cell counts accurately.

#### Time-lapse imaging and simulation of biased microscopic sorting

To evaluate the time requirements for scan imaging, 1 × 10^4^ cells were seeded. After 11 h, a total of 265 images were captured every 30 mins for 6 days. The data were plotted in graph format at 12 h intervals.

To understand the variation in cell density based on the observation position, a simulation was conducted. Sampling was performed from 25 fields of view, each of the same size as a typical microscopic image (using a field of view with a 4× lens on the Keyence BZ7000 microscope). The average cell count for each image was calculated to estimate the cell count for the entire dish. The discrepancy between the estimated and the actual cell counts was then determined. The data from a single field represented the time series changes in the estimated number of cells at each fixed point within the 25 fields of view. Data from 3 to 20 fields of view were processed by considering five combinations of sampling fields.

### Quantification and statistical analysis

All statistical analyses were conducted using GraphPad Prism 9 (GraphPad Software, La Jolla, CA, USA). The results are reported as mean ± SD or as the maximum (Max) and minimum (Min) values. For multiple comparisons, the Kruskal–Wallis test with Dunn's test was employed, with significance determined based on the value at NoSheet. Correlation analysis was conducted using Pearson's correlation coefficient. A significance level of P < 0.05 was considered statistically significant using two-tailed tests.

### Additional resources

This study is part of a clinical research and the clinical registry number is UMIN000033162. https://www.umin.ac.jp/ctr/index-j.htm.

## References

[1] Altman D.G. (1994). The scandal of poor medical research. BMJ.

[2] Kaiser J. (2016). If you fail to reproduce another scientist’s results, this journal wants to know. Science.

[3] Baker M. (2016). Biotech giant publishes failures to confirm high-profile science. Nature.

[4] Freedman L.P., Gibson M.C., Ethier S.P., Soule H.R., Neve R.M., Reid Y.A. (2015). Reproducibility: changing the policies and culture of cell line authentication. Nat. Methods.

[5] Goodman S.N., Fanelli D., Ioannidis J.P.A. (2016). What does research reproducibility mean?. Sci. Transl. Med..

[6] Begley C.G., Ioannidis J.P.A. (2015). Reproducibility in science: improving the standard for basic and preclinical research. Circ. Res..

[7] McGrath M., Tam E., Sladkova M., AlManaie A., Zimmer M., de Peppo G.M. (2019). GMP-compatible and xeno-free cultivation of mesenchymal progenitors derived from human-induced pluripotent stem cells. Stem Cell Res. Ther..

[8] Tirughana R., Metz M.Z., Li Z., Hall C., Hsu D., Beltzer J., Annala A.J., Oganesyan D., Gutova M., Aboody K.S. (2018). GMP production and scale-up of adherent neural stem cells with a quantum cell expansion system. Mol. Ther. Methods Clin. Dev..

[9] Lawrence M., Evans A., Moreau T., Bagnati M., Smart M., Hassan E., Hasan J., Pianella M., Kerby J., Ghevaert C. (2021). Process analysis of pluripotent stem cell differentiation to megakaryocytes to make platelets applying European GMP. NPJ Regen. Med..

[10] Xie Y., Liu W., Liu S., Wang L., Mu D., Cui Y., Cui Y., Wang B. (2020). The quality evaluation system establishment of mesenchymal stromal cells for cell-based therapy products. Stem Cell Res. Ther..

[11] Mizuno M., Endo K., Katano H., Amano N., Nomura M., Hasegawa Y., Ozeki N., Koga H., Takasu N., Ohara O. (2021). Transplantation of human autologous synovial mesenchymal stem cells with trisomy 7 into the knee joint and 5 years of follow-up. Stem Cells Transl. Med..

[12] Sekiya I., Katano H., Mizuno M., Koga H., Masumoto J., Tomita M., Ozeki N. (2021). Alterations in cartilage quantification before and after injections of mesenchymal stem cells into osteoarthritic knees. Sci. Rep..

[13] Kempner M.E., Felder R.A. (2016). A Review of Cell Culture Automation. JALA.

[14] Holland I., Davies J.A. (2020). Automation in the life science research laboratory. Front. Bioeng. Biotechnol..

[15] Suga M., Kii H., Niikura K., Kiyota Y., Furue M.K. (2015). Development of a monitoring method for nonlabeled human pluripotent stem cell growth by time-lapse image analysis. Stem Cells Transl. Med..

[16] Ince C., Ypey D.L., Diesselhoff-Den Dulk M.M., Visser J.A., De Vos A., Van Furth R. (1983). Micro-CO2-incubator for use on a microscope. J. Immunol. Methods.

[17] Schwartz S.D., Hubschman J.P., Heilwell G., Franco-Cardenas V., Pan C.K., Ostrick R.M., Mickunas E., Gay R., Klimanskaya I., Lanza R. (2012). Embryonic stem cell trials for macular degeneration: a preliminary report. Lancet.

[18] Baghbaderani B.A., Tian X., Neo B.H., Burkall A., Dimezzo T., Sierra G., Zeng X., Warren K., Kovarcik D.P., Fellner T., Rao M.S. (2015). cGMP-manufactured human induced pluripotent stem cells are available for pre-clinical and clinical applications. Stem Cell Rep..

[19] Molina-Ruiz F.J., Introna C., Bombau G., Galofre M., Canals J.M. (2022). Standardization of cell culture conditions and routine genomic screening under a quality management system leads to reduced genomic instability in hPSCs. Cells.

[20] Osaki T., Kageyama T., Shimazu Y., Mysnikova D., Takahashi S., Takimoto S., Fukuda J. (2017). Flatbed epi relief-contrast cellular monitoring system for stable cell culture. Sci. Rep..

[21] Kesavan S.V., Momey F., Cioni O., David-Watine B., Dubrulle N., Shorte S., Sulpice E., Freida D., Chalmond B., Dinten J.M. (2014). High-throughput monitoring of major cell functions by means of lensfree video microscopy. Sci. Rep..

[22] Pushkarsky I., Liu Y., Weaver W., Su T.W., Mudanyali O., Ozcan A., Di Carlo D. (2014). Automated single-cell motility analysis on a chip using lensfree microscopy. Sci. Rep..

[23] Jin D., Wong D., Li J., Luo Z., Guo Y., Liu B., Wu Q., Ho C.M., Fei P. (2015). Compact wireless microscope for in-situ time course study of large scale cell dynamics within an incubator. Sci. Rep..

[24] Gao P.F., Lei G., Huang C.Z. (2021). Dark-Field Microscopy: Recent Advances in Accurate Analysis and Emerging Applications. Anal. Chem..

[25] Elsinga G.E., van Oudheusden B.W., Scarano F., Watt D.W. (2003). Assessment and application of quantitative schlieren methods: Calibrated color schlieren and background oriented schlieren. Exp. Fluid.

[26] Curl C.L., Bellair C.J., Harris T., Allman B.E., Harris P.J., Stewart A.G., Roberts A., Nugent K.A., Delbridge L.M.D. (2005). Refractive index measurement in viable cells using quantitative phase-amplitude microscopy and confocal microscopy. Cytometry A..

[27] Doulgkeroglou M.-N., Di Nubila A., Niessing B., König N., Schmitt R.H., Damen J., Szilvassy S.J., Chang W., Csontos L., Louis S. (2020). Automation, monitoring, and standardization of cell product manufacturing. Front. Bioeng. Biotechnol..

[28] Fontaine M.J., Selogie E., Stroncek D., McKenna D., Szczepiorkowski Z.M., Takanashi M., Garritsen H., Girdlestone J., Reems J.A., Biomedical Excellence for Safer Transfusion BEST Collaborative (2020). Variations in novel cellular therapy products manufacturing. Cytotherapy.

[29] Mizutani M., Nakajima K., Kino-oka M. (2022). Approach of resource expenditure estimation toward mechanization in the manufacturing of cell-based products. Regen. Ther..

[30] Manzini P., Peli V., Rivera-Ordaz A., Budelli S., Barilani M., Lazzari L. (2022). Validation of an automated cell counting method for cGMP manufacturing of human induced pluripotent stem cells. Biotechnol. Rep..

[31] Gunetti M., Castiglia S., Rustichelli D., Mareschi K., Sanavio F., Muraro M., Signorino E., Castello L., Ferrero I., Fagioli F. (2012). Validation of analytical methods in GMP: the disposable Fast Read 102® device, an alternative practical approach for cell counting. J. Transl. Med..

[32] Fechine G.J.M., Rabello M.S., Souto Maior R., Catalani L.H. (2004). Surface characterization of photodegraded poly(ethylene terephthalate). The effect of ultraviolet absorbers. Polymer.

[33] Hoque M.T., Mahltig B. (2020). Realisation of polyester fabrics with low transmission for ultraviolet light. Color. Technol..

[34] Pojman J.A., Viner V., Binici B., Lavergne S., Winsper M., Golovaty D., Gross L. (2007). Snell's law of refraction observed in thermal frontal polymerization. Chaos.

[35] Rivera-Ortega U., Hernández-Gómez C.R., Vega-Torres G., Lopez-Medina M.E. (2019). Simple apparatus to calculate the refractive index of liquids based on Snell's law. Measurement.

[36] Mizuno M., Matsuzaki T., Ozeki N., Katano H., Koga H., Takebe T., Yoshikawa H.Y., Sekiya I. (2022). Cell membrane fluidity and ROS resistance define DMSO tolerance of cryopreserved synovial MSCs and HUVECs. Stem Cell Res. Ther..

[37] Zhu H., Khanna S.K. (2016). A Novel Transparent Glass Fiber-Reinforced Polymer Composite Interlayer for Blast-Resistant Windows. J. Eng. Mater. Technol..

[38] Li C.H., Lee C.K. (1993). Minimum cross entropy thresholding. Pattern Recogn..

[39] Pal N.R. (1996). On minimum cross-entropy thresholding. Pattern Recogn..

[40] Mizuno M., Katano H., Otabe K., Komori K., Kohno Y., Fujii S., Ozeki N., Horie M., Tsuji K., Koga H. (2017). Complete human serum maintains viability and chondrogenic potential of human synovial stem cells: suitable conditions for transplantation. Stem Cell Res. Ther..

