# Supplementary material for: Noninvasive total counting of cultured cells using a home-use scanner with a pattern sheet
Source: iScience. 2024 Feb 9;27(3):109170. doi: 10.1016/j.isci.2024.109170 (PMC10884908; doi:10.1016/j.isci.2024.109170)

**iScience, Volume *27***

## Supplemental information

# Noninvasive total counting of cultured cells using a home-use scanner with a pattern sheet

**Mitsuru Mizuno, Yoshitaka Maeda, Sho Sanami, Takahisa Matsuzaki, Hiroshi Y. Yoshikawa, Nobutake Ozeki, Hideyuki Koga, and Ichiro Sekiya**

## Supplementary Online Content

**Figure S1.** Imaging and cell recognition in each culture vessel, related to Figure 1. From left to right: six-well plates, 15 cm dish, T75 flask, and T225 flask. Image of the cell region (Region) is shown in pseudo color.

**Table S1.** Simulation model used for scan imaging, related to Figure 2.

**Figure S2.** Cell visualization range, related to Figure 4. (A) The cell visibility region appears adjacent to the color transition border of the switchover region. Cells were visualized as objects. (B) Theoretical coverage of each pattern assuming that the area covered by the pattern transition is 680 μm.

**Figure S3.** Visibility of scan imaging using microbeads, related to Figure 4. (A) Information volume map (IVM) visualized as objects from the scan images using 1 to 30 μm microbeads. (B) IVM value difference between the representative background and objects. Higher values lead to higher object visibility in the reconstructed images.

This supplementary material has been provided by the authors to give readers additional information about their work.

Figure S1. Imaging and cell recognition in each culture vessel, related to Figure 1. From left to right: six-well plates, 15 cm dish, T75 flask, and T225 flask. Image of the cell region (Region) is shown in pseudo color.

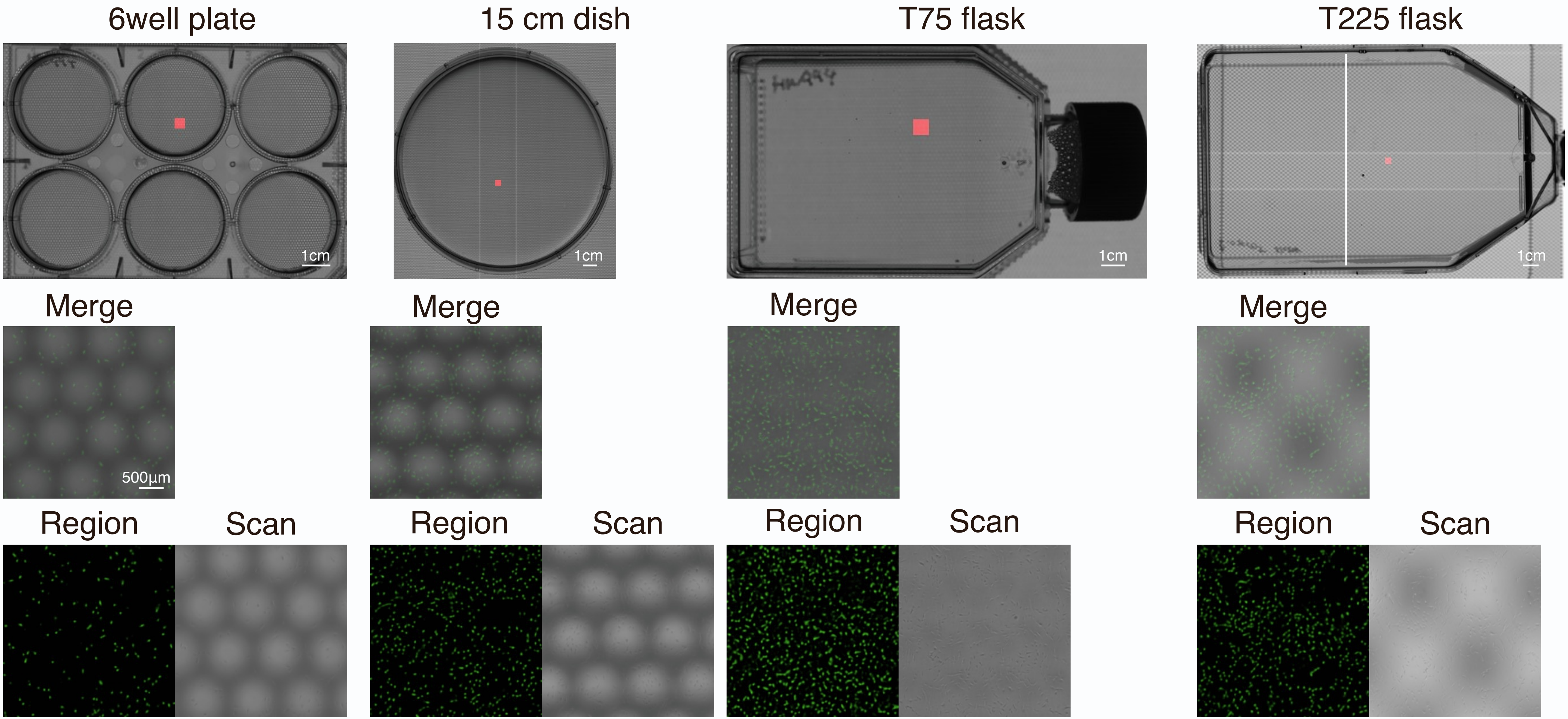

**Table S1.** Simulation model used for scan imaging**,** related to Figure 2.

| Label | Properties | | |
|---|---|---|---|
| Light source | White light-emitting diode (LED) (400–800 nm) | Complete diffusion | Surface emission |
| Pattern sheet | Diameter size: 200 µm | Hole pitch = 400 µm | Arrangement = Grid |
| Model cell | Long side 50 µm, Short side: 17.5 µm, height: 17.5 µm | Refractive index = 1.333 (589.3 nm) | Pitch = 10 × 10 |
| Lens | Magnification 1× | Curvature (lens 1) Front side: 101.4 mm, rear side: -18.9 mm | Curvature (lens 2) Front side: -18.9 mm, rear side 101.4 mm |
| Aperture | Circle 34 mm in diameter with a 15 mm hole | | |
| Photosensitive area | General charge-couple device | Total number of cells 250,000 | Cell size 4×4 µm |
| Generated number of rays | 500 million rays | | |

Figure S2. Cell visualization range, related to Figure 4. (A) The cell visibility region appears adjacent to the color transition border of the switchover region. Cells were visualized as objects. (B) Theoretical coverage of each pattern assuming that the area covered by the pattern transition is 680 μm.

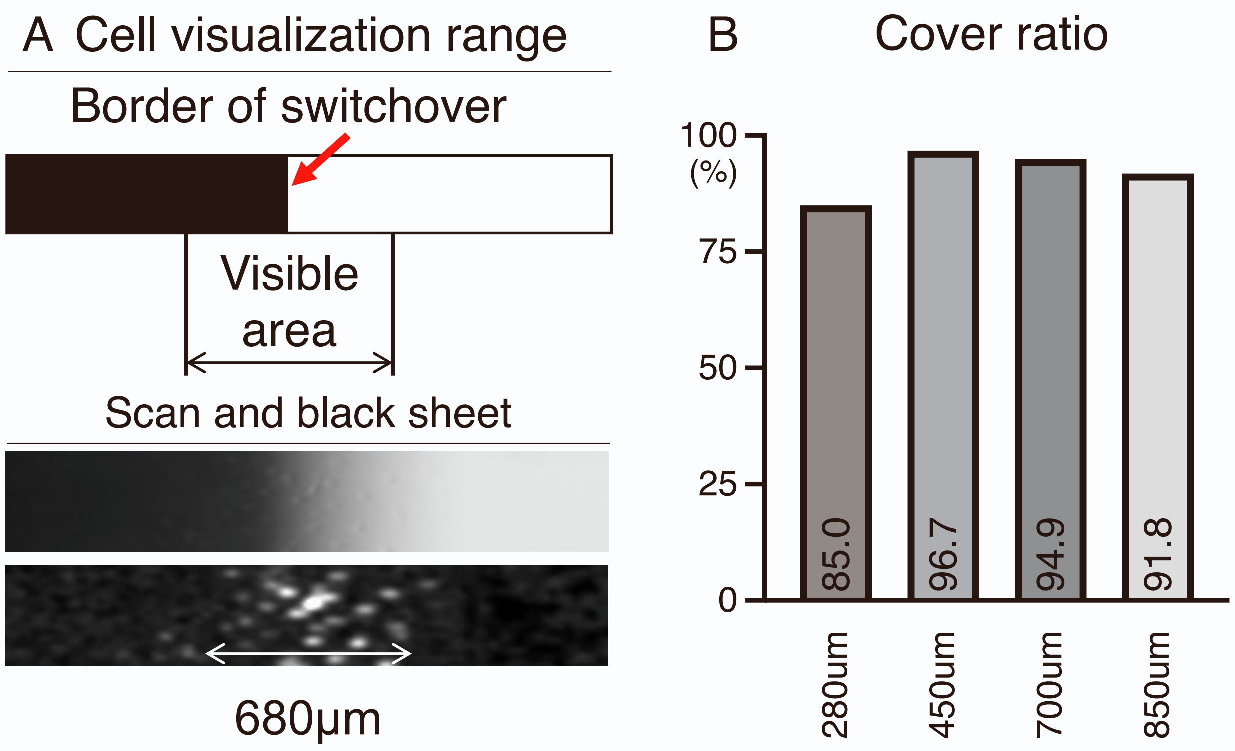

Figure S3. Visibility of scan imaging using microbeads, related to Figure 4. (A) Information volume map (IVM) visualized as objects from the scan images using 1 to 30 μm microbeads. (B) IVM value difference between the representative background and objects. Higher values lead to higher object visibility in the reconstructed images.

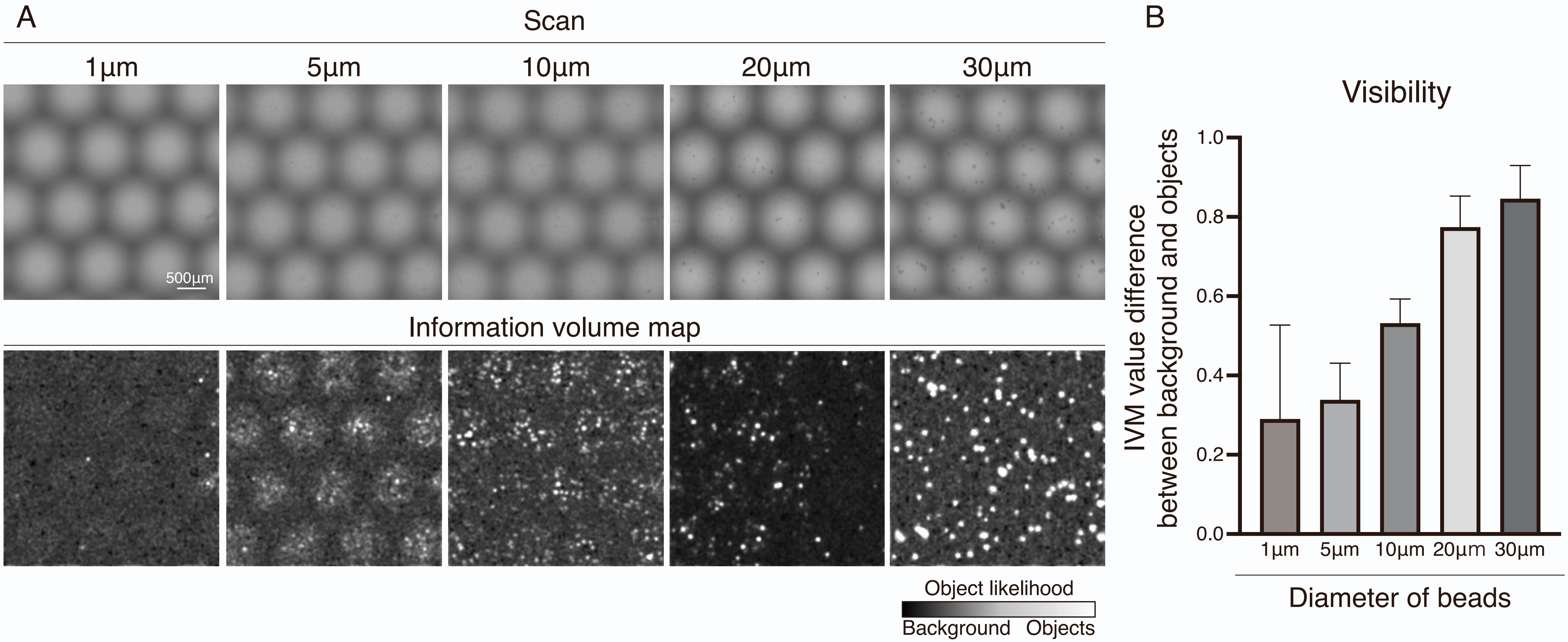

Supplement: Document S1. Figures S1–S3 and Table S1 [file mmc1.pdf]
